# Reducing the therapeutic vacuum: a qualitative study learning from experiences of care delivery during terror attacks in the UK over the past 20 years

**DOI:** 10.1136/bmjopen-2025-108881

**Published:** 2026-06-01

**Authors:** Timothy John Stephens, Dijay Dave, Amy Harriet Hughes, Benjamin Swift, Simon Markby Glasgow, Rachael Fothergill, Gareth Grier, Karim Brohi, Claire Louise Park

**Affiliations:** 1 Critical Care and Perioperative Medicine Research Group, Queen Mary University of London, London, UK; 2 Adult Critical Care Unit, Barts Health NHS Trust, London, UK; 3 Barts Health NHS Trust, London, UK; 4 Centre for Trauma Sciences, Queen Mary University of London, London, UK; 5 Forensic Pathology Service, Wantage, UK; 6 Imperial College Healthcare NHS Trust, London, UK; 7 London Ambulance Service NHS Trust, London, UK

**Keywords:** QUALITATIVE RESEARCH, ACCIDENT & EMERGENCY MEDICINE, Health policy

## Abstract

**Abstract:**

**Objectives:**

The complex and dynamic care context of terror attacks must be better understood to reduce deaths. This study was designed to understand the tension between saving lives and maximising safety for emergency responders attending active terror incidents.

**Design, setting and participants:**

Qualitative study exploring the experience of survivors and emergency responders (armed and unarmed police, paramedics, doctors and fire officers) present in the hot (unsafe) zone of five major terror attacks in the UK since 2000. We used reflexive thematic data analysis to build qualitative case studies, comparing similarities and tensions between perspectives of different participant groups.

**Results:**

In our analysis of over 2000 min of interview data from 26 participants, we found a common view that the priority during a terror-related mass casualty event was to save lives. However, responder groups maintained distinct mental models that shaped their operational priorities regarding treatment for those injured within the hot zone. All responders expressed willingness to take self-assessed risks to save lives, but better interagency communication was noted to be required to achieve this safely. All responders felt it was vital to have experienced health professionals present to triage and facilitate urgent treatment and extraction decisions. Armed police commanders had dual responsibilities to achieve rapid care delivery while preventing further terrorist-inflicted injuries. Operationally, this was perceived as leading to a lack of shared mental models between responders regarding what is ‘unsafe’ due to zoning, rather than communication of risk, potentially delaying vital care delivery. There were mixed survivor perspectives regarding the risks that responders should be exposed to, but broad agreement that there was a notable absence of health professionals present in the hot zone during the immediate aftermath of attacks.

**Conclusion:**

There is strong professional and public support for improving care delivery, including potential hot zone working, to minimise the therapeutic vacuum in active terrorist attacks. Better risk communication and better shared mental models are necessary to balance responder risk with care delivery to maximise lives saved as safely as possible.

**STRENGTHS AND LIMITATIONS OF THIS STUDY**

- Large, maximum variation sample of participants with experience of a range of terror attacks.
- Reflexive thematic analysis maximised opportunities to learn and to draw actionable findings from our detailed dataset.
- Focused on UK attacks only.
- Snowball sampling was necessary but may have limited sample selection.
- We acknowledge that protocols and training for such events have evolved since 2017.

## Background

 Violent attacks causing intentional fear and harm, whether motivated by terrorism or another ideology, can result in mass killing and have caused significant global morbidity and mortality.^1 2^ Such attacks are associated with a high number of prehospital fatalities and use a number of methods, including firearms, bladed weapons, improvised explosive devices, sieges, motor vehicles, fire and chemicals.^1 3^ In the UK, when mobile attackers motivated by terrorism are the perpetrators, these attacks are referred to as marauding terrorist attacks (MTAs) and refer to all methodologies of attack.^4^ Globally, in 2023, the total deaths caused by terrorism increased by 22% to 8352, the highest level since 2017. Even excluding the 7 October attacks in Israel, there was still an increase of 5% from 2022.^2^ Deaths are not the only impact, and the global economic impact of terrorism on the world economy through injury, property damage and loss of gross domestic product income has been estimated to be US$855 billion from 2000 to 2018.^5^

An optimal prehospital response to such an attack requires a rapid progression from first responder or life-saving interventions (eg, opening the airway) to bridging interventions (those required to stabilise and facilitate a rapid extrication) and enhanced care interventions (including blood products, ventilation, chest decompression and, if necessary, endovascular haemorrhage control).^6^ At the same time, there is a need to maintain the safety of all on-scene responders and the public from further injury or attack. In a UK context, this is achieved through the creation of zones—hot (unsafe), warm (potentially safe for responders with appropriate protective equipment and training) and cold (safe)—to demarcate operational boundaries.^3^ Acknowledging that emergency response systems and terminology vary internationally, similar challenges face all responders to such events, and similar lessons have repeatedly been identified.^7^ The lack of timely medical intervention, as highlighted in recent UK inquiries,^8 9^ has been previously termed the ‘therapeutic vacuum’,^10^ and more recently ‘The Care Gap’ by the Chairman of the Manchester Arena Inquiry in the UK.^9^ Outside of formal judicial inquiries, there has been to date very limited empirical research that explores the causes of this care gap from the perspective of those involved.^7^ Those with experience of directly delivering or receiving care during such attacks are limited, yet these few individuals have vital insights into the causes of this care gap.

This study is part of a larger UK National Institute of Health Research-funded research project focused on reducing the ‘therapeutic vacuum’ and therefore deaths from terror events in the UK and globally. The overall aim of this multiproject study is to determine whether some lives could be saved by trained personnel entering the ‘hot zone’ to deliver specific medical treatments to the casualties. To do this, we are investigating what causes people injured in such attacks to die prior to reaching hospital and in what timeframe (project 1: synthesis of existing published evidence and project 2: expert review of deaths occurring in terror and terror-like criminal events (eg, multivictim attacks)).^11 12^ This evidence will allow us to know if any of the medical interventions that are currently available could have stopped them from dying and what type of police officer, paramedic or doctor would be able to deliver the intervention. If we know how long they were alive for, we can work out when the medical intervention would need to happen in order to save their life (project 4: computer modelling of optimal response strategies). Any changes to care delivery or implementation of new interventions will require a deeper understanding of this dynamic and complex care context, including the barriers to timely delivery of care in higher threat zones, to facilitate the effective delivery of the outputs from the overall study. This qualitative study (project 3 in the overall study) is designed to explore the experience of a range of emergency responders (police, fire, paramedical and medical) and survivors of terror attacks on: (a) what working or being in the unsafe (hot) zone of a terror attack is like; (b) how care to the injured was delivered or not; and (c) the perceived facilitators of and barriers to care delivery.

## Methods

### Participation identification and recruitment

Between 2021 and 2023, we recruited via email people who had direct experience of working in and around the hot zone of a UK terrorist attack or were a survivor of an attack. A maximum variation sample of frontline professionals were recruited considering key characteristics (profession, seniority and terror event). A sample of survivors were recruited to include all UK terror events in the past 20 years. These five events comprise four in London—the 7 July 2005 (7/7) attacks, the Westminster Bridge (WB) attack in 2017, the London Bridge/Borough Market (LB/BM) attacks in 2017 and the Fishmongers’ Hall (FmH) attack in 2019—and one outside London, the Manchester Arena (MA) attack in 2017. Recruitment for professionals was through professional networks of the authors and snowball sampling,^13^ with initial recruits from professional networks approaching suitable colleagues about their willingness to be interviewed and providing them with the lead researcher’s (TJS) contact details. Recruitment for survivors was via support groups and networks, primarily Survivors Against Terror,^14^ with extensive support from lead members of that group (see the Acknowledgements section). We made a pragmatic estimate that a purposive sample of up to 30 participants would provide a sufficient degree of ‘data saturation’,^15^ being at or above the higher end of suggested sample sizes for qualitative research with focused objectives, accounting for variation in experiences between participants.^16 17^

### Data collection procedures and content

All data were collected via online interviews (using Microsoft Teams or Zoom) by two of the authors (TJS±CLP), except where interviewees were directly known to CLP, when TJS conducted the interview alone. Individual interviews were conducted for frontline professionals and small group interviews for survivors (up to a maximum of three participants). Interviews had two distinct parts: a narrative opening where the participant was asked to tell their story of the event from their perspective, and a second semistructured part that followed a set of open questions, based on the content of the narrative account and following an interview topic guide (see the onlinesupplemental files 1  2).^18^ We informed the interviewees that all information would be handled confidentially and published in an anonymous format. The interviewees consented to the recording and transcribing of the interviews, and we obtained explicit written consent for all verbatim quotes used and their tagging. Following specific guidance from a psychologist, we also started each interview with a structured ‘check-in’, acknowledging the traumatic nature of the event and reminding them it was entirely acceptable to pause or even completely stop the interview at any time. We also completed an interview ‘check-out’ to understand how the participant was feeling, what they would do if they started to have intrusive thoughts and who to contact if they wanted further support following the interview. The study protocol is available in the online supplemental material. In the protocol, we refer to data collection for survivors using ‘focus groups’; however, we elected to use the small group interview format to facilitate more in-depth data collection using narrative accounts while allowing for some (limited) peer interaction between participants.

### Interviewer and core study team member (TJS, DD, AHH and CLP) positionality

TJS is a critical care nurse and clinical lecturer, with specific skills in qualitative research. DD is a junior doctor who was undertaking a research rotation at the time of this study. Neither had any experience of, or substantive opinions about, the study topic or research objectives. AHH is a prehospital clinician and consultant in emergency medicine, who was involved in the hospital-based triage of patients injured in the LB/BM event. CLP is a consultant critical care and prehospital physician and is highly experienced in prehospital medicine both in the civilian and military world. CLP has clear views about the role of health professionals within the hot zone of a terror attack and as such was not involved in either coding or initial theming of these data.

### Data analysis

**Figure 1:**
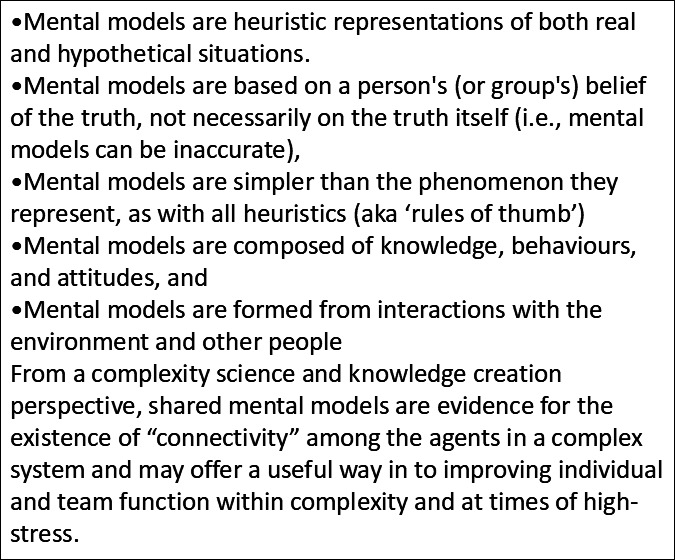
Brief summary of key features of a mental model.^20^

As this study was exploratory, an inductive and reflexive approach to thematic analysis was employed. Reflexive thematic analysis is a theoretically flexible interpretative approach to qualitative data analysis that facilitates the identification and analysis of patterns or themes in a given dataset.^15 18^ We used the following key steps (see also the online supplemental material): (1) each interview transcript was carefully read and re-read and then coded; (2) three of the same transcripts were coded by two authors (TJS and DD) and then an initial data meeting, and the first coding structure was agreed and established; (3) TJS and DD then continued to code independently, with further regular data meetings to check in on and revise the codes as necessary. From data meeting discussions it became apparent there were more similarities (in perspectives and thus codes) by professional role than there were by terror event. As such, as we commenced theming, a case study approach was employed, with transcripts grouped by profession, and codes being aggregated into case study themes for each professional group (eg, the ‘case of the paramedics perspective’).^19^ Key cross-cutting themes were developed reflexively from the cases to represent key high-level tensions and similarities across the large dataset. Here we were guided by the concept of mental model theory (ref 20; see also figure 1) to facilitate the summarising of varied perspectives. Three data meetings with the core author team (TJS, DD, AHH, CLP) were held throughout the process for TJS and DD to present, discuss and justify themes developed. The draft results were presented back to participants by email for review and comment in a reflexive member-checking exercise.^15^ The majority of participants gave feedback during this exercise (and all whose verbatim quotes were used), which led to minor alterations to the text only. Finally, the manuscript was reviewed by the overall study oversight committee and the patient and public involvement (PPI) group.

### Patient and public involvement

Our overall study PPI group consisted of four survivors of terror and three survivors of non-terror traumatic events, who have been involved from the outset of this study. They have assisted with participant information sheet wording, advice regarding how to sensitively approach potential interview participants as well as reviewing the findings and final manuscript prior to submission.

## Results

In total, 32 participants were invited, either through initial seeding via author networks or subsequent snowball approaches. We interviewed 26 participants in total (seven female, 19 male), with over 2000 min of audio interview. Interviews ranged from 67 min to 98 min in length, with an average length of 80 min. The breakdown of types of participants and the incidents is detailed in table 1. Six invitations were not responded to (one paramedic, one unarmed police officer, four survivors (four male, two female)).

**Table 1:** Study participants, roles of participant type during a terror situation and terrorist events sampled

Participant type	n	Roles during a terror situation
Unarmed police officer	4 (1 off duty)	To safely establish what is happening, direct and disperse potential victims, gain relevant information and provide updates to commanders, maintain and develop safe observations, contain subjects or limit their movement, or confront the threat where this is safe/achievable. They are not expected to endanger their own or their colleagues’ lives in doing so. Officers carry only batons, with some equipped with tasers, for personal protection. Although they often end up providing first aid while the area is being secured by armed police, this is not their primary role, and they had no formal role in casualty care or triage prior to 2023.
Armed police officer	2	To locate, confront and neutralise the threat, and to provide cover that enables other emergency services to enter a warm zone safely. They are able to operate in hot zones and are required to secure an area as warm to maximise the safety of other responders. They had no role in casualty care or triage before 2023.
Armed police commander	2	Otherwise known as the tactical firearms commander (TFC), this role serves as the ground-assigned commander of firearms assets at the scene. The TFC is responsible for multiagency discussions and decision-making at the forward command point to ascertain zones and tactics.
Paramedic	3	Two types: ‘specialist’ paramedics have ballistic protection and are able to enter the warm zone to triage and treat casualties; ‘non-specialist’ paramedics who since February 2019 have also been permitted to enter the warm zone but do not have ballistic protection and were not routinely allowed into these areas before that date. Neither group would knowingly be sent into a hot zone, although they may find themselves there and must then decide whether to stay.
Prehospital physician	1	Often referred to as enhanced care doctor, they act as medical advisers to the ambulance service and are responsible for triaging and treating the sickest patients. They are known as ‘Non-specialist’ responders, who prior to February 2019 were not routinely deployed into warm or hot zones, but have been technically permitted to enter these areas since February 2019 with appropriate training and personal protective equipment (PPE) for the threat.
Fire commander	1	To lead decision-making regarding the deployment of fire and rescue assets at the scene. They advise on fire and rescue-related matters and identify where the Fire and Rescue Service (FRS) can help within the scene (not just regarding fire, but building safety, extrication and assistance with casualty care).
Fire officer	2	May be tasked to enter the scene, including areas requiring ballistic protection if they are trained as specialist responders. Depending on fire conditions and air quality, they may or may not be operating in breathing apparatus.
Security officer	1	No official emergency service role or casualty treatment role. The role will depend on location and facility for whom they work.
Member of public	8	None—usually ushered out but may choose to stay and help.
Relative of victim	2	None—usually ushered out but may choose to stay and help.

Event	n	Brief description of the event
7/7	4	Four attackers wearing suicide bombs that were detonated at 4 attack sites (3 tubes and a bus) in central London just before and after 09:00 on 7 July 2005. There were 52 killed and over 770 reported injured.
Westminster Bridge (WB)	3	On 22 March 2017, a single attacker used a car as a weapon and drove over WB (from south to north) before crashing into the fence outside the palace of Westminster. Proceeded to run around and fatally stab a policeman. There were 5 killed and approximately 50 injured.
London Bridge/Borough Market (LB/BM)	10	On 3 June 2017, three attackers drove a van onto LB, hitting pedestrians before crashing into the railings outside the Barrow Boy and Banker pub. They then ran down the steps towards the Boro Bistro and through BM, armed with knives taped to their wrists and wearing what were later confirmed to be fake improvised explosive device (IED) vests, stabbing multiple people. There were 8 killed and approximately 48 injured.
Fishmongers’ Hall (FmH)	2	On 29 November 2019, a single attacker attending a ‘Learning together’ event at FmH attacked a number of other delegates with a knife while wearing what was later confirmed to be a fake IED vest. There were 2 killed and 3 injured.
Manchester Arena (MA)	6	On 22 May 2017, a single attacker detonated a suicide bomb (in a rucksack) in the City Room of MA as the Ariana Grande concert was ending. 22 people were killed and up to 1000 injured.

### Perspectives on delivering care in the hot zone

Our analysis found that the range of professionals coming together in a multiagency response to a terror attack had varied perspectives on their own, and their colleagues’, task priorities. As one participant put it:

When police go into a place they are looking at the bad guys and looking round. So they’re looking straight ahead for the bad people. The ambulance are looking at the floor at the injured people and when the fire go in, we’re looking at the structure of the building. We’re looking at the layout of it. What’s going on? (Fire officer, MA)

**Figure 2:**
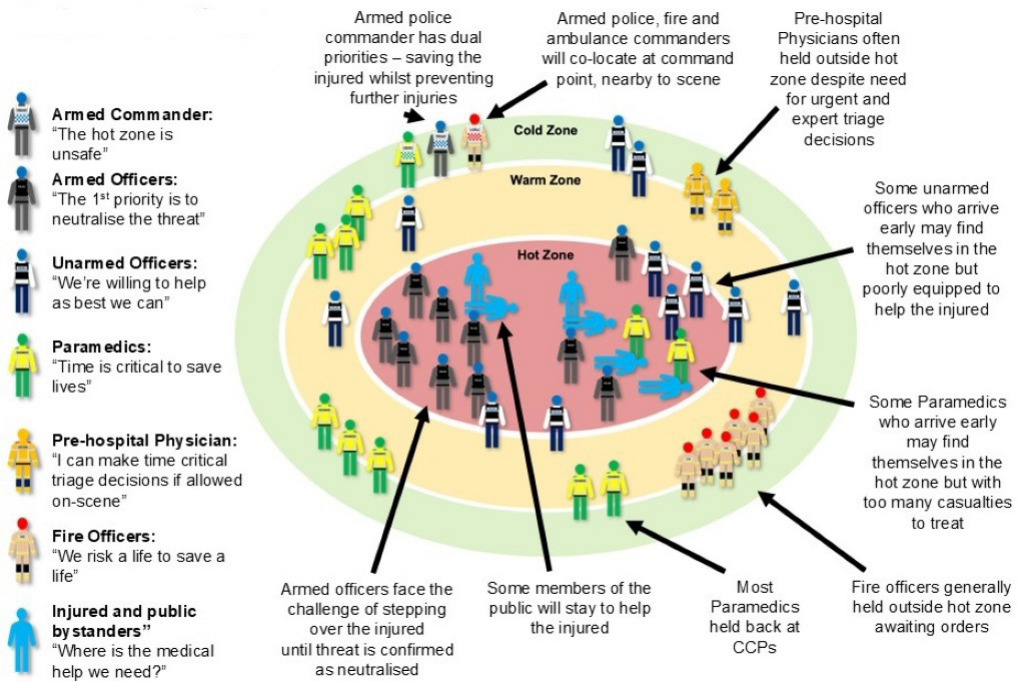
Actors, locations and mental models of responders. CCP, casualty clearing point.

Table 2 summarises the key commonly expressed perspectives of each professional group regarding providing care within the hot zone of a terror attack, and figure 2 displays the locations and deployment typical of the five attacks we studied. The similarities and tensions between these perspectives highlighted the challenges to, and some opportunities for, holding a collective view of how to deliver care to the injured in a hot zone context. We report these key cross-cutting similarities and tensions between these professional groups below.

**Table 2:** Summary of key perspectives on hot zone working by professional/role

Professional	Mental model	Key perspectives on hot zone working
Police commander (armed)	“The hot-zone is unsafe.”	Tension between treating injured and preventing further injuries. Hot zone is unsafe. The aim is to minimise hot zone as fast as possible. On-scene, dynamic leadership and direct communications with those who know what is happening are key. Prefer extraction to on-scene care wherever possible.
Police—armed	“The hot-zone is unsafe.”	Priority is neutralising threat. There is a moral challenge of stepping over injured. Want professional healthcare on scene as fast as possible (vs doing own care).
Police—unarmed ‘response’ officers	“We’re willing to help as best we can.”	Willing to be ‘in there’ to help as best we can. Trauma training and kit is considered poor. Need expert support to guide treatment and extraction decisions.
Prehospital physician	“Time is critical to saving lives.”	Time is critical to saving lives. Want to make own risk assessment regarding safety. Need to be near to patients to triage and make urgent extraction/treatment decisions.
Paramedic	“Time is critical to saving lives.”	If police and public are there, we should be too. Want to make own risk assessment regarding safety. Need to switch from single-patient to multipatient perspectives—this is hard. Early extraction is key.
Fire	“We risk a life to save a life.”	We risk a life to save a life. Why are marauding terrorist attacks (MTAs) so different from other mass casualty incidents? We are trained and ready to go. Leadership decisions regarding deployment are too procedure based.
Public	“Where is the help we need?”	It is a unique, horrific and life-changing experience. Medical/paramedical help often feels missing. Mixed views regarding the levels of risk healthcare professionals should be expected to be exposed to.

#### Cross-cutting themes: similarities

##### Theme 1: professionals are willing to take risks to help the injured

###### Entering or staying in the hot zone to help viewed as a difficult decision, but the ‘right’ one

The first key similarity, across all participants, was a willingness to enter and stay within the unsafe zone of a terror attack in order to help those injured. This was described by some as a sense of vocation and was common whether the participant had any specialist training, including for MTA, or not. This included an off-duty police officer present nearby to LB/BM who ran towards the event as everyone else was running away. One police officer who arrived very early on scene stated:

I heard the commotion, screaming and so I naturally—as you do as a copper—sort of run towards that. (Unarmed police officer, LB/BM)

Other non-police participants described decisions that were less instinctual and more deliberative as to whether to move forward or withdraw, but central to all these accounts was a strong feeling of vocation to help wherever possible. This feeling of vocation was also combined for some with feelings of fear, complicating the decision to stay and help or return to safety. The humanity of such decision-making was brought home by one participant who reported calling their father before proceeding further into the hot zone. They went on to say:

I had all these patients, and I knew some of them were dying and had died. Common sense said, “I need to get out of here.” But at the same time, I knew I couldn’t, because the police were staying there and there were members of the public also there. (Paramedic*,* LB/BM)

###### Risk, to varied extents, viewed as part of the daily work of an emergency responder

Other participants pointed out that, as emergency responders, they were experienced, to greater or lesser degrees, with assessment and management of risk in their daily working lives. Many described taking steps to protect themselves as they began to realise that they were within an unsafe zone of an MTA. Some participants had specialist training and equipment to work within a warm zone environment and so used this to provide additional safety as they found themselves in the hot zone. Others described the rapid risk assessment they undertook before moving forward:

I don’t want to die. I don’t want to get blown up. I don’t want any of my team to get harmed at all. But I’ve heard enough and I’ve seen enough to know that the vast majority of times we’re not actually taking a risk, you know, we know we’ve got a good gut instinct for what’s safe and what’s not. Now you got to balance it out with running in blindly and just getting shot or blown up or dragged into a second, you know, explosive scene or whatever. (Prehospital physician*,* FmH)

###### Emergency responders considered how to protect themselves while providing care

Participants discussed the actions they took to protect themselves and colleagues in undeniably challenging situations. Some participants already had specialist ballistic personal protective equipment due to their role, while others used more basic measures such as standing on watch for danger while colleagues provided care:

We were taking it in turns holding our batons, so we were doing obviously rounds of CPR and then taking it in turns, just sort of guarding each other, giving first aid…Again, because I know that we were massively on offer where we were, especially when that would be their [terrorists] route back to the van*. *(Unarmed police officer*,* LB/BM)

In sum, our participants, who all have direct experience of working in the unsafe zone of a terror attack, were unanimous in feeling that the decision to do so, while not always easy, was absolutely the right decision. It should be noted that while many were exposed to horrific scenes as a result of this decision, none were physically harmed.

### Theme 2: triage, urgent treatment and extraction of the injured is best led by health professionals wherever possible

#### Police are willing to help the injured but feel poorly trained and equipped

The second key similarity/cross-cutting theme was the view that care for the injured was best provided by health professionals. The extent to which this was viewed as possible within the unsafe zone, and how this might be achieved, did vary between the armed police and other groups of participants. This tension will be discussed below. What was apparent from the unarmed police officer participants was that their willingness to get in and help the injured was not matched by training and first aid kits that were unsuitable for the kind of injuries before them:

You can’t be fully trained to deal with an explosion. I know you can’t be that super duper trained, but I just felt really, really under-prepared. (Unarmed police officer*,* MA)

#### Armed police are more trauma trained than unarmed police officers but have other priorities

Armed police reported having superior training and equipment but also expressed a strong preference to having experienced healthcare professionals there to deal with the traumatic injuries on scene. This preference was in part because having expert help on scene would allow them to focus on their primary task, threat neutralisation, allied with acceptance that an experienced paramedic or prehospital physician would simply provide more effective care. The desire for police officers to have expert help was summed up by one prehospital physician, who described hesitating momentarily after a request from armed officers to follow them into an apparently active unsafe zone:

I think one of them looked at me and the fact that I didn’t immediately follow him in, he did swear or said something to that effect and was like, OK…., you know, he made a very quick, immediate decision. You’re either going come in, support us and take my word for it, or you’re going to go into that clipboard zone and start having meetings. (Prehospital physician*,* FmH)

Armed officers also noted the moral challenge of having such training but also having to walk past the injured until their primary role of neutralising the threat had been achieved.

It’s certainly hard for us, when we’re going forward, and you’ve got people asking for help, and you’re just having to turn around, “Oh, go round there and someone will help you.” (Armed police*,* LB/BM)

#### Agreement that priority in the hot zone should be triage and extract decisions

Extraction, rather than providing intensive care on scene, was viewed as a key priority by all participants. For the police, the expressed ideal would involve officers extracting patients to a casualty clearing point to be treated. (The interviews were carried out before triage was routinely taught to police officers in the UK.) Where this was not feasible, triage, basic rapid treatment and extraction by paramedics was viewed mostly as the safest option in dynamic risk situations that could change quickly, offering the best balance from their perspective between treating the injured and preventing further injuries:

I need paramedics behind me to stabilize any casualties, do what they can do, take the casualty and go. If they can’t take the casualty, then treat and leave. Unfortunately, in these incidents demand for their specialist skills will outstrip the resource. (Armed police commander*,* WB)

#### Health professionals feel there is a role for expert eyes on scene to make urgent decisions

For health professionals, the value of rapid extraction was centred around their knowledge that rapid and definitive treatment in a hospital often confers the greatest survival benefit for certain injuries, particularly penetrating injuries. Healthcare professionals saw a limited but clear role for experienced clinicians to be on scene (in the unsafe zone) to perform rapid intuitive triage and support urgent extraction decisions:

I think, the best you can do in the hot zone is to work in pairs to triage patients appropriately, to bounce decisions off each other, as in, to have a quick preliminary discussions, triage prioirty-1, priority-2, priority-3, and then not deliver interventions in a hot zone, rather extricate and deliver [those interventions] them in a warm or cold zone*.* (Paramedic, 7/7)

Not only is triage not in the skill set of many police officers but also there may be a (understandable) reluctance, due to the nature of the injuries, for many to make vital extraction decisions. Participants described the value in these circumstances of an expert eye, where clinical gestalt of intuitive triage enables rapid and potentially life-saving decision-making to occur. As one participant explained:

…they [police officers] were with her tending to her, trying to wrap her wounds in bandages and stuff like that, when I said, is she alive? They had to actually stop, stoop down and check if she was alive or not. To me, that means you’re critically injured. If someone has to stop and check if you’re alive. So that was worrying. And once they stopped and checked and said umm yes she’s alive. I said right, get her out… (Prehospital physician, FmH)

In sum, all our participants viewed the expertise of healthcare workers on scene as essential to meet the needs of the severely injured. How exactly this might be operationalised emerged as a tension between the participant groups and is covered in the following section.

### Cross-cutting themes: tensions

#### Theme 3: lack of shared mental models of what is ‘unsafe’ due to zoning rather than communication of risk

##### Armed police view is that the hot zone is completely unsafe but should be kept small

The first area of tension between cases centres around defining the genuinely unsafe area where no one apart from armed police should go. The police commander and armed police view seemed at first to provide a reasonable definition of the hot zone:

[a] hot-zone is about an area where you really just can’t be because there’s bullets flying all over the place and we’re going to lose paramedics and police officers in greater numbers than probably the casualties are already there. (Armed police commander, MA)

Further, in keeping with the careful balancing of their dual priorities to allow care delivery while preventing further injuries, minimising the boundaries of the hot zone was described as important to allow medical interventions to occur as expediently as possible:

And we also got reports going, that he was not alone when he crashed the car. The armed people have run up onto the bridge. So I was like right. We’ve got a warm zone. We’ve got a hot-zone right now. I need to reduce that hot-zone as quickly as possible. (Armed police commander, WB)

##### Other emergency responders feel that zoning prevents getting to the injured timeously

Yet, establishing where may or may not be safe in the early and highly dynamic stages was described as difficult, leading some professionals to rely more on their own judgement or professional instinct.

I’ve got that background knowledge of there’s someone in there dying. In fact, there might be loads of people in there dying and I could have just stood there and waited to be told it was safe. But if I’m honest, I think I would have stood there for absolutely ages… And if I know I’m not going to get that information quickly, I’m going to just start moving forward until I decide it’s not safe, right? (Prehospital physician, FmH)

Therefore, the tension around perspectives and priorities came out as somewhat fraught, especially as any incident like this will rely on interagency working, which in turn relies on shared mental models of what is going on and what can and cannot be done to save lives:

…my point of view, at the incident you’re not going to know what the hot-zone is and what the warm zone is. It’s just so dynamic, you’re not going to know. And I think to save lives you have to send people in, when you have the intelligence that there is a police presence there and there are patients dying. I do not think that is justifiable, holding clinicians back. [however] I don’t think I could send a clinician in with no police presence there, because that’s crazy. (Paramedic, LB/BM)

##### Experienced emergency responders feel able to make their own assessments of risk

Allied to this was a view expressed by some non-police participants that treating MTA as a radically different risk from other high-risk major incidents did not make complete sense, and that risk and its assessment and mitigation are part and parcel of the emergency responders’ working life. These participants talked soberly about the risks of their jobs and the strategies they employ regularly to minimise these as best they can.

As firefighters, we would get going into risk areas on a daily basis; it could be a fire with a gas cylinder involved, you know? Alright, a firearm is a little bit different but if you put controlling measures in place and make it as safe as possible, there’s no reason why people could not work within that environment safely, or as safe as could be. They could get harmed at a fire as well as this marauding type attack. (Fire commander, LB/BM)

##### Communicating risk versus blanket ‘zoning’ (ie, hot, warm and cold zones)

To solve this tension, participants expressed a preference for more detailed and clear information to be shared between actors early on scene during terror attacks. Rather than the terminology around hot, warm and cold, participants spoke of communication mirroring that used during other incidents, rapidly outlining what was known and not known about risks and allowing each professional group some greater autonomy about their preferred next step. One participant described what they wished had happened in the incident that they were involved in:

I think they should have been given very quick brief and it should have been told that we’ve got police officers, member of the public and [staff] in there, the protocol is we don’t go in until it’s declared safe. We can’t declare it’s safe at the minute because we were concentrating on casualties rather than searching. Do you want to go in? …These are the circumstances. This is what could happen. People are totally forgiven if you say no, I don’t want to go in. Everyone wants to go home at the end of the day. Course we do. But at least give them the option of going. (Unarmed police officer, MA)

In sum, there was a tension between the armed police and other emergency responders’ views about the optimal way to deliver rapid and effective care to the injured during a terror attack. This tension is at first understandable given the dual priorities of the police during such incidents compared with the more singular role of the other emergency workers. However, the accounts of emergency responders about their daily risk assessment and mitigation experience and how this may still be of substantial relevance to terror attack incidents revealed a more complex tension, whereby the autonomy of these workers is reduced in the name of safety. The need to provide safety of course remains a priority, but how this is done was called into question by many of those with direct experience of such events.

What prevents us from going into a hot-zone and what prevents us from treating people in a hot-zone? Well, the problem is calling it a hot-zone in the first place. (Fire officer, MA)

### The survivors’ perspective

A common perspective among survivors was just how surreal and chaotic these situations are, upending their view of normal life and often having long-lasting psychological impact:

I’d still say there’s times where I kind of think if it actually happened or if I’m just making it up completely…it was a very surreal experience to be surrounded by a whole host of guns and knives and big bangs at the time…what I think of as the most poignant and something that stuck with me the most is just seeing people get injured, is something that was so, so out of the ordinary. (Survivor, LB/BM)

#### Broad agreement that the lack of healthcare professionals on scene was notable

Within these chaotic situations, many participants reported their surprise at the lack of help for those injured from emergency services, at least in the early stages:

Yeah, but you do [expect help], you see it on these films, you see it in movies, you expect people to come in. There was no one. No one came in. It was members of the public that were helping. (Survivor, MA)

Many participants also reported that care was being delivered by other members of the public to those injured and an overall sense of camaraderie among those in the hot zone:

I think it was just bystanders…I remember at one point when we were standing around, a policeman ran up and said “Is anybody here a doctor?” and somebody said “Well, I used to be a paramedic but I haven’t been for a couple of years,” and the policeman shouted “Good enough,” and he went off with this guy. (Survivor, LB/BM)

#### Divided opinions about the level of risk emergency responders should be exposed to

In terms of hot zone working, survivors were somewhat divided on whether healthcare professionals should be actively working on the unsafe zone or not. Some held views that security should come first, as it does currently, and that you could not fault responders not wanting to take risks. Others, however, felt it should be part of the role of the emergency services to help injured no matter what:

You know, as a kid growing up, the emergency services were someone that rushed. They rushed in. The firefighters rushed in, the ambulance. The police, they all rushing and you know, since this happened, all I’ve seen is the emergency services stand. Standing away. You know and members of the public rushing in you know and that’s sad for me you know. (Survivor, MA)

## Discussion

Regarding our study aims, we have described, from multiple perspectives, what it is like to be in or work in the hot zone of a terror attack. For professionals entering or staying in the hot zone to help the injured was viewed as a difficult decision, but the ‘right’ one due to a strong sense of vocation. Professionals talked about using their risk assessment skills to judge if they were ‘safe enough’ to stay and provide treatment. Regarding how care was delivered, for both professionals and survivors, the lack of more help arriving for the injured was reported as a notable feature of their experiences. Non-healthcare professionals reported providing what care they could with their often limited training. Healthcare professionals described the limits to what care they could provide without further human resources. Survivors described care being provided to the injured by other members of the public. Barriers to the delivery of care were viewed as resulting mainly from the lack of timely access to patients due to the lack of clear shared mental models around aims and zoning. In addition, poor communication of risk contributed by delaying timely and effective decisions to send responders forward or change zoning. Regarding facilitators of care delivery, there was broad agreement that triage, treatment and extraction decisions are best led by experienced health professionals where possible. In particular, delivery of simple, life-saving interventions and extracting the right patients in a timely fashion were viewed as key to optimising survival. There was agreement that more timely decisions and response would be facilitated by clearer shared mental models between agencies and the enhanced passage of risk information, especially in the early stages where there is the greatest potential to save lives.

Our study adds to the limited number of qualitative studies designed to learn from the experiences of emergency responders involved in, and survivors of, terrorist attacks.^7^ A study on care delivery during the 2011 Norway attack^21^ found that ‘success factors’ included themes of ‘empowerment through multiprofessional networks that trusted each other’s autonomy’ and ‘the ability to improvise based on competence’. A study on the 2016 Belgian attack noted a chaotic care environment in the early stages of an attack and the moral challenges when receiving orders to stop or delay treating the injured due to perceived ongoing risks.^22^ A study on decision-making during critical and disaster incidents also identified the challenge commanders face in balancing saving the injured versus preventing further injuries but noted this high cognitive task load, especially at times of high uncertainty, could easily lead to ‘decision inertia’ and a failure to enact active operational decisions.^23^ A Swedish study expanded on our theme that responders are willing to take self-assessed risks by adding that greater levels of experience, either individually or of the crew overall, was linked to risk appetite, ability to maintain situational awareness and ability to take effective protective measures.^24^ Two mixed-methods studies focused on UK attacks (primarily the MA attack) and the Boston Marathon attack identified differences from our study in that clear communication, interteam collaboration and flexible leadership were found to be response facilitators.^25 26^ However, primarily only hospital-based health professionals participated (rather than from emergency responder groups). These two studies, like ours, highlighted the importance of shared team mental models, but their findings suggest that these are potentially easier to achieve within professional groups (even across institutions) versus interagency working and within more structured contexts such as hospital emergency departments versus active terror event scenes.

### Meaning of the study: possible explanations and implications for clinicians and policymakers

There is good evidence that reducing time to definitive treatment for penetrating trauma improves patient outcomes.^27 28^ However, during high-threat incidents with a high degree of uncertainty, decision makers (eg, police, fire or ambulance commanders) may make more conservative decisions in their efforts to balance risk/benefit of reducing the hot zone.^23 29^ Based on the evidence from multiagency responders, in our study and during UK judicial enquiries,^8 9^ a contributing factor to the repeated failures of the UK Joint Emergency Service Interoperability Principles (JESIP)^3^ is the lack of shared mental model of risk assessment, communication and mitigation between responding agencies. The contradiction in the public/survivor perspective highlights the challenges policymakers face regarding facilitating hot zone working. We suggest replacing the blanket delineation of hot, warm and cold zones with clear passage of risk information to those responders arriving on scene, improving situational awareness and providing the necessary contextualisation and explanation of the risk that supports effective decision-making. This would provide the opportunity for autonomous deployment rather than the responsibility of a police commander to ‘define a safe zone’ for others and therefore the associated delay or decision inertia to keep them safe. It also facilitates the expedition to ‘expert professional medical eyes on scene’ to facilitate rapid extraction of those patients most at risk.

At the time of these interviews, triage was not in the skill set of many police officers; however, the Ten Second Triage^30^ is now trained to all emergency services in the UK. However, there is still a need to differentiate between the sickest priority 1 patients requiring the most urgent extraction, and this is (understandably) a difficult and inappropriate decision for non-healthcare professionals. Thus, the critical value in these circumstances of an expert eye, where the clinical gestalt of intuitive triage enables rapid and potentially life-saving decision-making to occur, remains. This has implications for JESIP education going forward. In particular, the importance of all emergency services really understanding the position from which the other services are coming from (their mental models) could be added to current single agency training. In addition, focused implementation of this understanding within multiagency command and live training exercises could significantly impact the achievement of the JESIP principles.

Despite changes in UK response protocols, our findings are still highly relevant, as despite previously restricted access to higher threat zones, all participants experienced working in such areas. This also highlights that in the early stages of a terror attack, emergency responders may find themselves in zones of higher threat than commanders would intentionally place them, but they feel equipped to make appropriate risk benefit decisions in such circumstances.

### Unanswered questions and future research

Future research should include using existing evidence and decision-making theory to design and robustly evaluate interagency MTA preparedness to optimise their effectiveness in achieving clear risk communication and shared mental models between emergency responders. This micro-level preparedness, supporting frontline responders and commanders to optimise their decision-making under high pressure, has been found to be lacking.^29^ Further work would also be required to optimise the effective dissemination of learning throughout networks for all emergency responders (eg, unarmed police officers and non-specialist paramedics who may be first on scene in such attacks). An unanswered question relates to the benefit of fully embedding clinicians within the tactical police response in bridging the care and command gaps in the hot zone, enhancing tactical and medical information and therefore casualty flow from the hot zone to definitive care. This model has been shown to be effective in a number of countries, including France, Scandinavian nations, Austria, Hungary and a number of states across the USA. It was also recommended in a federal evaluation of lessons identified for improving responses to terrorist attacks in Germany, and the recent Manchester Area Inquiry further recommended a UK policing review of the evidence.^9 31^ However, introducing and operationalising this in some countries such as the UK requires further research, including potentially economic evaluation of the cost-benefit of such a model.

### Strengths and limitations

This study has several strengths, including its large, maximum variation sample of participants with experience of a range of terror attacks, the use of a narrative approach to data collection and reflexive thematic analysis to maximise opportunities to learn and to draw actionable findings from this detailed dataset. We adhered to accepted guidelines for ensuring trustworthiness of the research,^15 17^ including data triangulation, using in-line indicative quotes, a member checking exercise and efforts to maintain reflexivity throughout. Limitations include the focus on UK attacks only, the use of snowball sampling, which raises the risk of ‘groupthink’ by the way networks were used to recruit participants, and a slightly historical perspective when we should acknowledge that MTA protocols and training have evolved since 2017. We only recruited one prehospital physician whose experience might not be representative of all. We were also unable to recruit any medical or paramedical professionals from the MA attack, which we accept creates a lacuna in the perspectives offered on that event.

## Conclusion

There is strong professional and public support for improving care delivery, including hot zone working, to minimise the therapeutic vacuum and to maximise the ability to save lives during future terror attacks. Concerns remain regarding how to safely operationalise this. Given the chaotic nature of such events, further evidence-based preparation and training for emergency responders is urgently needed. This should aim to create better shared mental models through more effective risk communication and teach how to optimise balancing risk with delivering care to the severely injured.

## Supplementary material

10.1136/bmjopen-2025-108881 online supplemental file 1

10.1136/bmjopen-2025-108881 online supplemental file 2

10.1136/bmjopen-2025-108881 online supplemental file 3

10.1136/bmjopen-2025-108881 online supplemental file 4

## Data Availability

No data are available.
