# Supplementary material for: Reducing the therapeutic vacuum: a qualitative study learning from experiences of care delivery during terror attacks in the UK over the past 20 years
Source: BMJ Open. 2026 Jun 1;16(6):e108881. doi: 10.1136/bmjopen-2025-108881 (PMC13239495; doi:10.1136/bmjopen-2025-108881)
Supplement: online supplemental file 1 [file bmjopen-16-6-s001.pdf]

Interview topic guide: Frontline professionals

- Re-confirm consent to participate
- Confirm consent to record

Below are interview prompts. However, as this is predominately a narrative interview the key is to capture the participants story (narrative) and experience, the key events from their point of view and their thoughts and perspectives reflecting on these.

1. Describe briefly the study question and aims
2. Remind the participant that they can stop the interview at any time if they feel distressed
3. Ask the participant to tell them a little about themselves including exactly what their job role is, how long in their role and what is their role specifically during a terrorist attack
4. Ask the participant to describe their experience of working during a terrorist attack NB – *reassure the participant they don't need to provide precise times but rather paint a picture of what happened, the main events and their main thoughts / actions and their experience of all this*
    a. What was it like?
    b. What did you do? It may be helpful to describe a summary timeline of events from the participants perspective
    c. How did you feel? Initial reactions and also later
    d. What was your experience of providing care to injured people?
    e. Are there other important things you'd like share about the experience

Following this, more structured enquires will prompt discussion on the acceptability and feasibility of delivering specific healthcare interventions in a hot-zone environment.

These to include:

5. Ask the participants about, or otherwise, willingness to enter the hot-zone during a future terrorist attack, or (2) to allow teams to enter if in a command role, and the participants feelings and perceptions that shape this;
6. Ask the participant about mitigating factors that may affect the participants willingness to enter the hot zone, and strategies that could reduce perceived barriers to allow healthcare teams to enter.
7. Provide a list of potential life-saving interventions and explore the perspectives of the participant on the feasibility and acceptability of delivering these in the hot zone
    a. How could it work?
    b. Who would be doing these?
    c. What support would they need?
    d. What changes in practice or procedure may be required?

Check if the participant has any other thoughts related to study question they wish to share.
