# Supplementary material for: Reducing the therapeutic vacuum: a qualitative study learning from experiences of care delivery during terror attacks in the UK over the past 20 years
Source: BMJ Open. 2026 Jun 1;16(6):e108881. doi: 10.1136/bmjopen-2025-108881 (PMC13239495; doi:10.1136/bmjopen-2025-108881)
Supplement: online supplemental file 2 [file bmjopen-16-6-s002.pdf]

Focus group draft topic guide: members of the public with lived experience of a terror attack

- Re-confirm consent to participate
- Confirm consent to record

1. Describe briefly the study question and aims

Aim: to understand the acceptability and feasibility of delivering life-saving interventions in the hot-zone (unsafe zone) during a terror-attack situation, from the perspective of frontline professionals and the public.

Study Question 1:
What are the perspectives and views of frontline professionals, senior operational leaders and the public about working in the hot-zone (unsafe zone) during a terrorist attack situation?

Study Question 2:
What interventions (e.g. CPR (resuscitation, staunching bleeding, pain relief) would frontline professionals, operational leaders and the public consider as potentially acceptable, feasible and necessary to deliver in the hot-zone (unsafe zone) during a terrorist attack situation ?

2. Remind the participants that they can stop the focus group at any time if they feel distressed

3. Ask the participant to each tell a little about themselves e.g. job, where they live etc
4. Ask the participants to describe in turn their experiences of being involved or injured during a terrorist attack

    a. What was it like? Consider using up to 3 words to describe this and then ask for explanations…
    b. What happened? It may be helpful to describe a summary timeline of events from the participants perspective
    c. How did you feel? Initial reactions and also later as time went on
    d. **What was your experience of being given care? Are there things you would suggest to improve this for the future?**

5. Share some of the emergent findings about working in the hot zone from the frontline professional interviews with the group.
    a. Ask the group for their thoughts on these and any other perceptions they have about health workers and frontline responders delivering healthcare interventions in the hot zone

6. Are there other important things you'd like share about the experience?
7. Close focus group, stop recording
8. Debrief and time to talk if desired before
