# Supplementary material for: Reducing the therapeutic vacuum: a qualitative study learning from experiences of care delivery during terror attacks in the UK over the past 20 years
Source: BMJ Open. 2026 Jun 1;16(6):e108881. doi: 10.1136/bmjopen-2025-108881 (PMC13239495; doi:10.1136/bmjopen-2025-108881)
Supplement: online supplemental file 3 [file bmjopen-16-6-s003.pdf]

Joint Research Management Office (JRMO) Research Protocol for Research Studies

**Full Title**	Working in the hot zone: Perspectives of frontline professionals and the public on delivering care to the injured in the hot zone (unsafe zone) during a terrorist attack
**Short Title**	Working in the hot-zone study
**Sponsor**	▪ Barts Health NHS Trust (Barts Health)
**IRAS Number**<br>***Integrated Research Application System***	*n/a*
**Sponsor (EDGE) Number**	*<Sponsor's EDGE number>*
**REC Reference**<br>***Research Ethics Committee***	*<REC Reference number>*
**Chief Investigator (CI)**	Tim Stephens

1. Contents

1. Contents 2
2. Glossary 4
3. Signature page 5
4. Summary and synopsis 7
5. Introduction 8
   5.1. Background .... **Error! Bookmark not defined.**
   5.2. Rationale .... **Error! Bookmark not defined.**
   5.3. Risks / benefits .... **Error! Bookmark not defined.**
6. Study objectives 9
   6.1. Primary objective 9
   6.2. Secondary objective .... **Error! Bookmark not defined.**
   6.3. Primary endpoint .... **Error! Bookmark not defined.**
   6.4. Secondary endpoint .... **Error! Bookmark not defined.**
7. Study population 10
   7.1. Inclusion criteria 11
   7.2. Exclusion criteria 11
8. Study design 11
9. Study procedures .... **Error! Bookmark not defined.**
10. Statistical considerations .... **Error! Bookmark not defined.**
   10.1. Sample size .... **Error! Bookmark not defined.**
   10.2. Method of analysis .... **Error! Bookmark not defined.**
11. Ethics 12
   11.1. Annual Safety Reporting 13
12. Public involvement 13
13. Data handling and record keeping 13
   13.1. Data management 13
   13.2. Source Data 13

13.3. Confidentiality 13
13.4. Record retention and archiving 14
15. Safety reporting 14
16. Monitoring and auditing 14
17. Study committees 14
18. Finance and funding 14
19. Insurance and indemnity 14
20. Dissemination of research findings 15
21. References 15

2. Glossary

<Please insert any abbreviations and key terms>

3. Signature page

<DELETE AS APPLICABLE>

<Signature Agreement Option 1 (CI takes responsibility for statistics)>

CI Agreement

The study, as detailed within this Research Protocol, will be conducted in accordance with the principles of Good Clinical Practice (GCP), the UK Policy Framework for Health and Social Care Research, and the Declaration of Helsinki and any other applicable regulations. I agree to take responsibility for the statistical analysis and oversight of this study.

CI Name: **Tim Stephens**

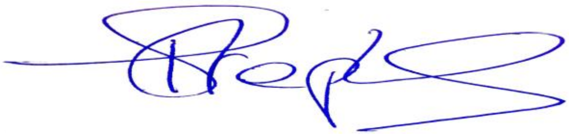

Signature:

Date: **08-06-21**

<OR>

<Signature Agreement Option 2 (separate statistician). This option should be used if the study involves randomisation (including batch allocation) or a cohort study of n=1000+ participants>

CI Agreement

The study, as detailed within this Research Protocol, will be conducted in accordance with the principles of GCP , the UK Policy Framework for Health and Social Care Research, and the Declaration of Helsinki and any other applicable regulations. I delegate responsibility for the statistical analysis and oversight to a qualified statistician (see declaration below).

CIname: ____________________

Signature: ____________________

Date: ____________________

Statistician's Agreement

The study as detailed within this research protocol will be conducted in accordance with the current UK Policy Framework for Health and Social Care Research,, the World

Medical Association Declaration of Helsinki (1996), Principles of ICH E6-GCP, ICH E9 - Statistical principles for Clinical Trials and ICH E10 - Choice of Control Groups.

I take responsibility for the statistical work in this protocol is accurate and take responsibility for statistical analysis and oversight in this study.

Statistician's name: ____________________

Signature: ____________________

Date: ____________________

4. Summary and synopsis

**Short title**	Working in the hot zone study
**Methodology**	Qualitative: interviews and focus groups only
**Objectives / aims**	To understand the acceptability and feasibility of delivering life-saving interventions in a terror-attack situation, from the perspective of pre-hospital providers and the public.
**Number of participants**	Professional: up to 30 pre-hospital healthcare workers, police and firefighters<br>Public: up to 32 members of the public
**Inclusion and exclusion criteria**	Inclusion criteria:<br><br>Maximum variation sample to include:<br>1. Paramedics, Pre-hospital Physicians, Police Officers, and Firefighters) in London and Manchester. Sampling will ensure an adequate variation in age, gender, years of professional experience, lived experience of working during a terrorist attack and seniority (to include both a range of ranks of frontline professionals and senior operational leaders).<br>2. People with lived experience of being involved in such an attack in London or Manchester.<br><br>Exclusion criteria:<br><br>• Unwilling or unable to give consent<br>• Inability to understand written and/ or verbal English
**Statistical methodology and analysis (if applicable)**	Inductive analysis using thematic and comparative analysis of interview and focus group transcripts
**Study duration**	12 months

5. Introduction

During the initial phase of terrorist events, a 'hot zone' is created where there may be on-going terrorist activities with active threats to life. Current policy is that emergency medical services are not allowed into the hot zone until it has been cleared, a process which may take hours. This is especially likely in marauding attacks, where there are multiple terrorists moving from location to location, such as in recent attacks in London and Paris, and active shooter events in the USA. The vast majority of deaths in these events occur at scene, before emergency services are allowed in the hot zone. Autopsy evidence from mass shootings in the US suggests that some patients die from potentially survivable injuries that may have been treated at scene [Smith et al, 2019]. If these findings are consistent across other types of events and healthcare systems, providing trauma care in the hot zone could dramatically reduce deaths in terrorist events.

However, to send emergency personnel into an area where there is an active terrorist threat to their own life is clearly a major decision. Potential gains from hot zone deployment must greatly offset the associated risks. Key information on which to make this decision does not currently exist, namely: (A) what proportion of prehospital terrorist deaths are potentially preventable; (B) What interventions would be required to prevent these; (C) how quickly these would need to be delivered; (D) who can deliver these interventions. Furthermore, it is important to understand whether frontline and command staff would perceive that therapeutic gains from hot zone deployment would justify the potential risks. These factors will vary depending on the nature and scale of each event and need to be synthesized into a system-level view of these events to allow balanced evidence-led decisions.

The potentially preventable causes of death in a terrorist attack depend on the mechanism. Available literature is predominantly military in origin, with haemorrhage as the leading cause in 90.9% of cases, and 8% associated with airway compromise.[Eastridge et al, 2012] Emerging civilian data has looked at causes of death in US active shooter events, where lung injury, was the most common cause of potentially preventable death. More data is required, but skills to manage pneumothoraces and ventilatory failure may be required forward of the cold zone where it is currently provided in the UK [https://www.jesip.org.uk/doctrine].

New Joint Operating Procedures that came into place this year now allow healthcare resources to operate forward of the cold zone, however training and operating procedures are yet to be developed to evolve the current response to allow this to occur, and there maybe barriers to commanders and responders being willing to do so which are extremely important to explore.

The timing of interventions is also critical if they are to have the potential to save life. Minimal data is available on exact timings of deterioration and intervention. Although dated, the most detailed database available is that of the Wound and Data Munitions Effectiveness Team (WDMET). This is meticulously collected data from 7,801 American casualties from Vietnam, documenting all causes, injuries, timings and interventions. In order to address the preventable deaths amongst the 42% that die in

the first 30 minutes (26% in 5 minutes and 16% in 5 to 30 minutes) [Carey, 1987], the interventions need to be performed by those who can rapidly access the casualties.

During the 2017 London Bridge terror attack, some casualties in the hot zone could not be accessed by the ambulance service for more than 3 hours due to them being designated a ‘hot zone’. These casualties were managed by police during this time. In his Regulation 28 report on action to prevent future deaths [Lucraft, 2019], the Chief Coroner noted that systems and practices should be examined to address the problem that hot and warm zones pose - that they could be designated for long periods of time, whole areas could be considered out of bounds for ambulance staff, and there is risk of delay in getting medical help to casualties.

Practice varies internationally, and in the 2015 Bataclan siege in Paris, two RAID doctors entered with police cover and triaged all (approximately 100) casualties from the orchestra pit while the intervention plan was co-ordinated [Crit care, 2016: 20(37)]. All of the live casualties were evacuated within 30 mins of RAID entering the theatre, and 30 mins before the terrorists were neutralised. Had the evacuation waited until after the police intervention, as in the UK, it would also have been 3 hours before any medical care was delivered. Only 1.4% of casualties evacuated from the Bataclan later succumbed to their injuries in contrast to approximately 10% in historical data, although the prehospital death rate remained high [Langlois & Ploquin, 2016].

This qualitative study presented for your review is part of a larger, NIHR funded, multimodal study to fill the identified evidence gap. The overall study will establish critical information on prehospital deaths and life changing injuries in terrorist events, their preventability, time course of deterioration and which therapeutic interventions would be required.

This qualitative study has been designed to explore the potential impact of hot zone working on different prehospital providers, from a range of perspectives, and the potential feasibility and acceptability of delivering therapeutic interventions in the hot zone to reduce overall mortality of future events.

6. Study objectives

6.1. Primary objective

To understand the acceptability and feasibility of delivering life-saving interventions in the hot-zone (unsafe zone) during a terror-attack situation, from the perspectives of emergency healthcare workers, other frontline professionals (fire and police) and the public.

Study Question 1:

What are the perspectives and views of emergency healthcare workers, other frontline professionals (fire and police), senior operational leaders and the public about pre-hospital providers working in a terrorist attack situation?

Study Question 2:

What interventions would emergency healthcare workers, other frontline professionals (fire and police) and operational leaders consider as potentially acceptable and feasible to deliver in a terrorist attack situation ?

7. Study population

We will recruit participants with lived experience of terror attacks in the UK. There are two specific groups we wish to recruit:

1. Healthcare professionals and other frontline workers, who have worked during such an event. N.B. With these participants we are seeking to understand their personal experience and views, rather than a representation of their employers views.
2. Members of the public who have been involved in such an event or potentially the relatives of those who have died during such an event.

We will purposively sample interview participants from two UK cities (London and Manchester) that have been the target for multiple terrorist attacks to provide variation of context. We will use different methods to recruit these participants.

Health professional and other frontline workers
We will recruit a maximum-variation, purposive sample of up to 30 interview participants, from the four main professional groups involved in providing a response to terrorist attacks (Paramedics, Pre-hospital Physicians, Police Officers, and Firefighters), across the two cities. Sampling will ensure an adequate variation in age, gender, years of professional experience, lived experience of working during a terrorist attack and seniority (to include both a range of ranks of frontline professionals and senior operational leaders).

Given the purposive sample and relatively small number of participants who meet our criteria we will use our research group professional networks and a snowball sampling approach. In this approach, contacts well known to the research group will be invited (by email) to be participants (with the ability to decline as per any research study). Following this, we will discuss our sampling requirements with these participants (referred to as ‘seeds’ in the literature) and ask if they know of any suitable colleagues who they could pass the invitation on to. We will continue to recruit using this approach until theoretical saturation is achieved (see also Data Analysis section below).

To protect potential participants from any coercion to join (e.g. by a manager or senior colleague) we will take the following steps:

1. We will only require 2-3 operational leaders to be participants. We will approach these directly using the professional networks of our group. Moreover, we will NOT ask these participants to act as seeds for further participants thus preventing any senior leader coercion.
2. For frontline professionals (paramedics, physicians, police and fire) we will a) impress upon the initial seeds the importance of the voluntary nature of recruitment and discuss with them who they may ask to participate, who and

why and b) as with any study, on initial contact with new potential participants we will give people clear opportunities to consider participation and ensure they understand they do not need to participate and can withdraw consent at any time prior to publication.

Members of the public with lived experience of terror attacks
We will advertise the opportunity to be a participant in this study through two known terror survivor groups with which our study group has contact. These groups are: The Survivors against Terror Group (https://survivorsagainstterror.org.uk) and the Counter Terrorist Association Network which has links to a group of survivors as well.

The invitation to participate and the Participant Information Sheet (PIS) will be sent by email by our contact in the group (who is either a family liaison officer or psychologist). These contacts will carefully consider who to invite, based upon knowledge of these groups and will approach the matter sensitively. Those who reply to this expressing interest will given the opportunity to ask further questions before agreeing or declining to participate.

7.1. Inclusion criteria

Maximum variation sample to include:

- Paramedics, Pre-hospital Physicians, Police Officers, and Firefighters) in London and Manchester. Sampling will ensure an adequate variation in age, gender, years of professional experience, lived experience of working during a terrorist attack and seniority (to include both a range of ranks of frontline professionals and senior operational leaders).
- People with lived experience of being involved in such an attack in London or Manchester.

7.2. Exclusion criteria

- Unwilling or unable to give consent
- Inability to understand written and/ or verbal English

8. Study design

Qualitative study design.

Health professional and other frontline workers

Semi-structured interviews will be conducted by the Principal Investigator (TS) by telephone / online using Zoom.

The interviews will start with narrative approach to encourage participants to relate their experiences and perceptions about working during a terrorist attack (in cold, warm or hot-zones) in depth. Following this, more structured enquires will prompt discussion on the acceptability and feasibility of delivering specific healthcare interventions in a hot-zone environment. These to include: (1) an exploration of the participants willingness, or otherwise, to enter the hot-zone during a future terrorist attack, or (2) to allow teams to enter if in a command role, and the participants feelings

and perceptions that shape this; (3) mitigating factors that may affect the participants willingness to enter the hot zone, and (4) strategies that could reduce perceived barriers to allow teams to enter. Interviews will be approximately 45-60 minutes in duration, will be audio recorded (with consent) and transcribed. Recruitment to interviews will continue until theoretical saturation is reached (maximum 30).

Members of the public with lived experience of terror attacks
Focus groups will be conducted by the Principal Investigator (TS) online using Zoom. The interviews will start with narrative approach to encourage participants to relate their experiences and perceptions of the event they were involved in. Following this, the concept of health workers and frontline workers delivering healthcare interventions will be explored. Focus groups will be approximately 60 minutes in duration, will be audio recorded (with consent) and transcribed. Recruitment will continue until theoretical saturation is reached (maximum 4 groups, 32 participants).

For both data sources, analysis will follow an inductive approach, with thematic and comparative analysis used to identify and develop key themes emerging from this rich dataset. Analysis will start after the 3rd interview and then continue concurrently with data collection. Data analysis will be led by the PI (TS) with regular data meetings convened with the study management team and PPI committee, to review and comment on findings. Analysis will use the software Nvivo (v.11) to collate and organise the data.

9. Ethics

Ethical approval is being sought from QMUL REC.
As this is a non-interventional qualitative study, it is low-risk in most respects but we are alive to the fact that the topic may be sensitive for potential participants.

The following risks, burdens and benefits are possible:

- Risks and burdens: participants may become distressed through the recounting of their experiences, either during the research activity or subsequently
- Benefits: participants are contributing to research that will have the potential to save the lives of people in the event of any future terror attacks and so are contributing something important to society by sharing their experiences and perceptions.

We address this sensitivity, and the risks and burdens, in the following ways:

1. Recruitment is through professional networks or through email advert and so there is no direct contact with the research team unless the potential participant makes contact
2. Once contact has been made, a PIS will be shared by a method of their choice (email or post) and then, again, the potential participant will be free to contact us if willing to participate or not if they do not wish to. We will send one single follow up to those who have expressed an interest but have not responded further. After this, no further contact will be made from the research team
3. Informed consent will be obtained for each participant and participants will be clearly made aware that they are free to cease participation at any time.

4. The interviews and focus groups will be led by the PI who is an experienced clinician and qualitative researcher, who will remain aware throughout of the need for sensitivity and look for any signs of participant distress
5. If any participant does show signs of distress the interview or focus group will be paused and the participant invited to either cease completely or temporarily their participation
6. For healthcare workers and other frontline professionals relevant support services will be signposted at the end of each interview.
7. For members of the public we will ensure that they have access to a support group including a clinical psychologist who already provides support to the groups from which we are recruiting .
8. All participants can be assured of anonymity and no identifying details will be used in any publications or other public forums (e.g. conferences).

9.1. Annual Safety Reporting

The CI will send an Annual Progress Report to the REC and the sponsor on the anniversary of the REC favourable opinion.

10. Public involvement

We have sought the views of key stakeholders – both professionals and members of the public who could have been potentially eligible participants to understand the acceptability of our research design and approach. They have confirmed the value of this study and its acceptability. Multiple suggestions to improve the language used in participant materials were offered and have been actioned.

We are in the process of convening a professional stakeholder group and a separate PPI group, who will meeting 6 monthly (virtually) to provide important input on study design, interpretation of findings and, latterly, in producing outputs for dissemination to meet a range of audience needs.

11. Data handling and record keeping

11.1. Data management

Data storage: Data collection and storage will follow the Barts Health / QMUL data protection policy (e.g. records of personal details (names, addresses, telephone numbers) kept in a secure place and separately from research records). All data will de-identified with raw data stored on a non-networked, password protected laptop along with a 'key' (i.e. a list of ID numbers and names) which connects the identifiable and de-identified data. Potentially identifiable material will not be used in published or publicly accessible outputs unless express written consent has been given (e.g. permission to use direct quotes will be sought.)

11.2. Source Data

Recordings of focus groups and interviews
Transcripts of focus groups and interviews

11.3. Confidentiality

Confidentiality and anonymity: digital recordings will be logged so that they will be identified with a participant number (or site number) only and all data are reported anonymously. Furthermore, participants will be reassured that any responses they give will not be attributed directly to them; if a direct quote is used in any research output, permission will be sought first and the quote not attributed directly.

11.4. Record retention and archiving

The data will be archived for 5 years in accordance with local standards and QMUL procedures for quality & assurance. Accessed by members of the research study team.

12. Safety reporting

Due to the nature and design of this study, safety reporting of adverse events will not occur.

13. Monitoring and auditing

The Sponsor or delegate retains the right to audit any study, study site or central facility. In addition, any part of the study may be audited by the funders where applicable.

14. Study committees

Our overall study, of which this qualitative research project is one sub-study, has a Study Management Oversight Committee comprising two senior clinicians, a clinical researcher and professionals from both the fire and police service, along with two co-chairs of the PPI group.

In addition there will be two PPI committees, one consisting of patients and public and professionals and one for patients and the public only, that will allow patients to feel able to freely share their views without the presence of professionals.

15. Finance and funding

This study is funded by the NIHR RfPB programme, grant number NIHR201453

16. Insurance and indemnity

The insurance that Queen Mary has in place provides cover for the design and management of the study as well as "No Fault Compensation" for participants, which provides an indemnity to participants for negligent and non-negligent harm.

17. Dissemination of research findings

We also plan to disseminate the findings and outputs of this project through a variety of mediums. A dissemination and communication plan will be co-produced with the PPI group and with support from the communications teams within Barts Health NHS Trust, the C4TS and LAA. Dissemination pathways will include academic research publications, conference presentations, network and association newsletters, media releases where appropriate, and on the London Trauma System and related websites.

In addition, through the applicants and collaborators on our research team we have access to networks of providers, leaders and policy makers related to emergency preparedness both across the UK and internationally. Specifically, members of our team provide leadership in the delivery of the UK National Interoperability Liaison Officer course. We intend to leverage both the formal and informal aspects of these networks to share the outputs of the project.

18. References

Smith ER, Sarani B, Shapiro G, etal . Incidence and cause of potentially preventable death after civilian public mass shooting in the US. J Am Coll Surg 2019;229:244-51. 10.1016/j.jamcollsurg.2019.04.016 31029762

Eastridge BJ, Mabry RL, Seguin P, etal . Death on the battlefield (2001-2011): implications for the future of combat casualty care. J Trauma Acute Care Surg 2012;73(Suppl 5):S431-7. 10.1097/TA.0b013e3182755dcc 23192066

Joint Emergency Services Interoperability Principles (JESIP). Joint doctrine: the interoperability framework (2nd ed). 2 Jul 2016. https://www.jesip.org.uk/joint-doctrine.

Wound Data and Munitions Effectiveness Team. The WDMET Study. 1970. Original data are in the possession of the Uni- formed Services University of the Health Sciences, Bethesda, Md. 20814-4799. Three summary volumes contain extensive abstracts of the statistical data and can be obtained from Defence Documentation Centre, Cameron Station, Alexandria, VA 22304-6145.

Michael E Carey. Learning From Traditional Combat Mortality and Morbidity Data Used in the Evaluation of Combat Medical Care. Military Medicine, Volume 152, Issue 1, January 1987, Pages 6–13.

HH Judge Lucraft QC, Chief Coroner of England and Wales. Inquests arising from the deaths of the London Bridge and Borough Market Terror Attack of 3 June 2017; Regulation 28 report on action to prevent future deaths. 1 November 2019. (https://londonbridgeinquests.independent.gov.uk/wp-content/uploads/2019/11/Final-Report-on-Action-to-Prevent-Future-Deaths-Report.pdf)

Service Médical du RAID. Tactical emergency medicine: lessons from Paris marauding terrorist attack. Crit Care 2016;20:37. 10.1186/s13054-016-1202-z 26872946

Langlois and Ploquin. Medecin du Raid: Vivre en etat d’urgence. Edition Albin Michel 2016.

This protocol is based on JRMO Protocol template for Research Studies; V3.0 01.03.2021
