# Supplementary material for: Reducing the therapeutic vacuum: a qualitative study learning from experiences of care delivery during terror attacks in the UK over the past 20 years
Source: BMJ Open. 2026 Jun 1;16(6):e108881. doi: 10.1136/bmjopen-2025-108881 (PMC13239495; doi:10.1136/bmjopen-2025-108881)
Supplement: online supplemental file 4 [file bmjopen-16-6-s004.docx]

| **Key steps in qualitative analysis process** | |
| --- | --- |
| Stage | Key Steps |
| Reading and familiarisation | - Each interview transcript was carefully read and re-read. - Notes and memos made. |
| Coding | - Three of the same transcripts were coded by two authors (TS and DD) and - Initial data meeting - Initial coding structure was agreed and established |
|  | - TS and DD then continued to code independently, - Regular data meetings to check in on and revise the codes as necessary. |
| Theming | - Three data meetings with the core author team (TS, DD, AH, CP) were held throughout the process for TS and DD to present, discuss and justify themes developed. - From data meeting discussions it became apparent there were more similarities (in perspectives and thus codes) by professional role than there were by terror event. - As such, as we commenced theming, a case study approach was employed, with transcripts grouped by profession, and codes being aggregated into case-study themes for each professional group (e.g. the ‘case of the paramedics perspective’). |
|  | - Key, cross-cutting themes were developed reflexively from the cases, to represent key high-level tensions and similarities across the large data-set. |
| Theory introduced | - During theming we were guided by the concept of mental model theory to facilitate the summarising of varied perspectives. |
| Member checking | - The draft results were presented back to participants by email for review and comment in a reflexive member-checking exercise. - The majority of participants gave feedback during this exercise (and all whose verbatim quotes were used), which led to minor alterations to the text only. |
| Final review | - Finally, the manuscript was reviewed by the overall study oversight committee and the Patient and Public Involvement group. |
