# Supplementary material for: Reducing the therapeutic vacuum: a qualitative study learning from experiences of care delivery during terror attacks in the UK over the past 20 years
Source: BMJ Open. 2026 Jun 1;16(6):e108881. doi: 10.1136/bmjopen-2025-108881 (PMC13239495; doi:10.1136/bmjopen-2025-108881)
Supplement: Supplementary file 5 [file bmjopen-2025-108881.reviewer_comments.pdf]

PEER REVIEW HISTORY

BMJ Open publishes all reviews undertaken for accepted manuscripts. Reviewers are asked to complete a checklist review form and are provided with free text boxes to elaborate on their assessment. These free text comments are reproduced below.

ARTICLE DETAILS

Title (Provisional)

Reducing the therapeutic vacuum: a qualitative study learning from experiences of care delivery during terror attacks in the United Kingdom over the past 20 years

Authors

Stephens, Timothy John; Dave, Dijay; Hughes, Amy Harriet; Swift, Benjamin; Glasgow, Simon Markby; Fothergill, Rachael; Grier, Gareth; Brohi, Karim; Park, Claire Louise

---

VERSION 1 - REVIEW

**Reviewer**	**1**
**Name**	**Davis, Joshua**
**Affiliation**	**Vituity, Emergency Medicine**
**Date**	**31-Aug-2025**
**COI**	**None**

Thank you for allowing me to review this qualitative study on health care during terror attacks in UK. I will start this review by clearly stating I am not a qualitative methods expert and this study should be reviewed by one. I think overall it is an important topic and the study adds valuable insights. I think there is some attention to detail that is needed before it is ready for publication. I provide some detailed comments for improvement below.

Title: acceptable

Abstract:

Well done.

Strength and limitations section is missing key limitations, particularly sample selection limitations

Introduction/Background:

Very well done and situates readers to UK context well.

Violent attacks "utilize a number of methods", not "methodologies" (which is the study of methods)

Several commas, including Oxford comma, are missing in the last sentence of the first paragraph and throughout the manuscript.

Methods:

"a maximum variation" seems to be unnecessarily repeated as it applied to both professionals and survivors.

should say "the lead researcher's"

First sentence of "data collection procedure and content" is a run on.

"checked in with the participant" is not detailed enough academically or readers to be able to replicate the findings.

Again, Oxford comma is missing and confusing.

Kudos to the authors for excluding the author with pre-established opinions on the topic and their detailed approach (and description of said approach) to prevent bias

The sentences: "They will help us in the writing of Plain English summaries of our findings, to be disseminated across various media formats" are not clear in relation to the manuscript and appeared to be copied from elsewhere.

Results:

First sentence could be reworded for clarity

Overall, good analysis and summary

Would recommend avoiding the controversial and derogatory term "providers" except in instances where used by participants.

Demographic characteristics of participants (vs. non participants) is notably missing.

Discussion:

Oxford comma missing often but inconsistently.

Generalizability is limited by analysis of characteristics of participants vs. non participants.

Author contributions does not clarify who drafted the manuscript.

References care not cited in a consistent manner, including journal article capitalization and DOI formatting.

I did check 5 references for accuracy ad found them to be cited correctly unless noted above.

Table/Figures

Each table/figure needs a title that is detailed enough to stand alone and a legend for any abbreviations used within each figur eor table for the same reason.

Reviewer 2

Name **Holmström, Inger K.**

Affiliation **Mälardalens universitet, School of health, care and social wlefare**

Date **03-Dec-2025**

COI **None**

Overall comments:

Thank you for the opportunity to read and comment on this manuscript. The study explores a range of emergency responders experiences of being in the hot zone during a terror attack in the UK, how care was delivered during the attack and facilitators or barriers for care delivery. This is an interesting and indeed important topic for both practitioners' and researchers, and the manuscript has a clear news value and is topical. The manuscript is well-written and fits well into the aims and scope of BMJ Open. I have only some minor comments, please see below.

Title, abstract and key-words

The title is descriptive and well chosen. The abstract is informative and well-written. The keywords well chosen. Maybe "UK" could be added to the keywords, as practice may vary between countries?

Introduction and literature-review

This section is well-written and use up-to-date international references. The study rationale is clearly described.

Methods

The sampling method is justified given the circumstances. It is impressive to have conducted a total of 2000 minutes of interviewing. I would, however, like to know the mean interview time for participants, an also the minimum och maximum interview time (range). It is good to know that you got support from a psychologist in conducting these interviews about traumatic experiences.

The concept of "lived experiences" is used frequently in the manuscript, but there is no theoretical elaboration regarding this. Why not use the concept "experiences" instead, as you do not delve into the theories of researching lived experiences? Or, if you would like to take the other way around this, add some text about researching lived experiences. See e.g van Manen's textbook "Researching lived experiences"

The data analysis is described in text, but I would also like to see the steps in the analysis displayed in a table of figures, to enhance trustworthiness.

Findings

The themes and sub- themes are well described and supported with a range of quotes which enhances confirmability.

Discussion and conclusions

The discussion is interesting, and the results are contrasted to previous research. I suggest that some implications for education are incorporated in this section.

The limitations could preferably include references and discussion in relation to quality criteria for qualitative studies.

VERSION 1 - AUTHOR RESPONSE

Reviewer: 1
Dr. Joshua Davis, Vituity
Comments to the Author:
Thank you for allowing me to review this qualitative study on health care during terror attacks in UK. I will start this review by clearly stating I am not a qualitative methods expert and this study should be reviewed by one. I think overall it is an important topic and the study adds valuable insights. I think there is some attention to detail that is needed before it is ready for publication. I provide some detailed comments for improvement below.

Title: acceptable

- Abstract:
Well done.

Strength and limitations section is missing key limitations, particularly sample selection limitations

Response: We have now added the following:

“Snowball sampling was necessary but may have limited sample selection” as you suggest

Introduction/Background:
Very well done and situates readers to UK context well.
Violent attacks "utilize a number of methods", not "methodologies" (which is the study of methods)

Response: Thank you. We have revised as you suggest.

Several commas, including Oxford comma, are missing in the last sentence of the first paragraph and throughout the manuscript.

Response: We have added an oxford comma and also simplified this sentence thus:

Deaths are not the only impact and the global economic impact of terrorism on the world economy through injury, property damage, and loss of Gross Domestic Product (GDP) income ~~as well as deaths~~ has been estimated to be US$ 855 billion from 2000 to 2018.[5]

Methods:
"a maximum variation" seems to be unnecessarily repeated as it applied to both professionals and survivors.

Response: The repetition has been removed.

should say "the lead researcher's"

Response: The apostrophe has been added.

First sentence of "data collection procedure and content" is a run on.

Response: This has been rectified.

"checked in with the participant" is not detailed enough academically or readers to be able to replicate the findings.

Response: We already specifically describe what the check-in comprised in the text, which would allow for replication of process:

"…acknowledging the traumatic nature of the event and reminding them it was entirely acceptable to pause or even completely stop the interview at any time."

However, we have amended the wording slightly to indicate this was a structured 'check-in' process, as recommended by our clinical psychologist advisor.

Again, Oxford comma is missing and confusing.

Response: We have added.

Kudos to the authors for excluding the author with pre-established opinions on the topic and their detailed approach (and description of said approach) to prevent bias

Thank you.

The sentences: "They will help us in the writing of Plain English summaries of our findings, to be disseminated across various media formats" are not clear in relation to the manuscript and appeared to be copied from elsewhere.

Response: We have deleted this sentence.

Results:
First sentence could be reworded for clarity

Response: We have revised the sentence thus:

In total, 32 participants were invited, either through initial seeding via author networks or subsequent snowball approaches.

Overall, good analysis and summary

Thank you.

Would recommend avoiding the controversial and derogatory term "providers" except in instances where used by participants.

Response: We have replaced this with the word professionals.

Demographic characteristics of participants (vs. non participants) is notably missing.

Response: The key characteristics that we sampled by were profession, seniority, and terror event. We have noted these in Table 1 by the participants. Apart from sex, no other characteristics or demographics were collected so as to minimise the amount of data collected from participants. We do however already state that non-response was from 'one paramedic, one unarmed police officer, four survivors' so the reader can understand the profession and seniority of the two professional non-responders. We have now added the breakdown of non-response by sex too:

"(one paramedic, one unarmed police officer, four survivors *(four male, two female)"*

Discussion:
Oxford comma missing often but inconsistently.
Response: We have gone through and checked for this.

Generalizability is limited by analysis of characteristics of participants vs. non participants.

Response: See above responses and edit to the responder / non-responder issue

Author contributions does not clarify who drafted the manuscript.
Response: This has now been added.

References care not cited in a consistent manner, including journal article capitalization and DOI formatting.
I did check 5 references for accuracy ad found them to be cited correctly unless noted above.

Response: Thank you. We have cheeked all our references for accuracy.

Table/Figures
Each table/figure needs a title that is detailed enough to stand alone and a legend for any abbreviations used within each figure or table for the same reason.

Response: We have amended the titles as you suggest and ensured all abbreviations are explained in the table or in a legend

Reviewer: 2
Dr. Inger K. Holmström, Mälardalens universitet, Uppsala Universitet

Comments to the Author:

Overall comments:
Thank you for the opportunity to read and comment on this manuscript. The study explores a range of emergency responders experiences of being in the hot zone during a terror attack in the UK, how care was delivered during the attack and facilitators or barriers for care delivery. This is an interesting and indeed important topic for both practitioners' and researchers, and the manuscript has a clear news value and is topical. The manuscript is well-written and fits well into the aims and scope of BMJ Open. I have only some minor comments, please see below.

Title, abstract and key-words
The title is descriptive and well chosen. The abstract is informative and well-written. The keywords well chosen. Maybe "UK" could be added to the keywords, as practice may vary between countries?

Response: Thank you, this has been added.

Introduction and literature-review
This section is well-written and use up-to-date international references. The study rationale is clearly described.

Thank you.

Methods
The sampling method is justified given the circumstances. It is impressive to have conducted a total of 2000 minutes of interviewing. I would, however, like to know the mean interview time for participants, an also the minimum och maximum interview time (range).

Response: Thank you. We have added the following :

Interviews ranged from between 67 to 98 minutes in length, with an average length of 80 minutes.

It is good to know that you got support from a psychologist in conducting these interviews about traumatic experiences.

Thank you.

The concept of "lived experiences" is used frequently in the manuscript, but there is no theoretical elaboration regarding this. Why not use the concept "experiences" instead, as you do not delve into the theories of researching lived experiences? Or, if you would like to take the other way around this, add some text about researching lived experiences. See e.g van Manen's textbook "Researching lived experiences"

Response: In the interests of simplicity we have edited the manuscript to just use the word / concept of experience rather than "lived experience."

The data analysis is described in text, but I would also like to see the steps in the analysis displayed in a table of figures, to enhance trustworthiness.

Response: We have now created a table of the key analysis steps as a supplementary file.

Findings

The themes and sub- themes are well described and supported with a range of quotes which enhances confirmability.

Thank you.

Discussion and conclusions

The discussion is interesting, and the results are contrasted to previous research. I suggest that some implications for education are incorporated in this section.

Response: *Thank you. We have added some text about training implications to the end of existing implications for clinicians and policymakers section,*

This has implications for JESIP education going forwards. In particular, the importance of all emergency services really understanding the position from which the other services are coming from (their mental models) could be added to current single agency training. In addition, focussed implementation of this understanding within multi-agency command and live training exercises could significantly impact the achievement of the JESIP principles.

The limitations could preferably include references and discussion in relation to quality criteria for qualitative studies.

Response: We have added the following text to the limitations sections,

We adhered to accepted guidelines for ensuring trustworthiness of the research,[13, 15] including data triangulation, using inline indicative quotes, a member checking exercise and efforts to maintain reflexivity throughout.

VERSION 2 - REVIEW

**Reviewer**	**2**
**Name**	**Holmström, Inger K.**
**Affiliation**	**Mälardalens universitet, School of health, care and social**
**wlefare**	
**Date**	**14-Apr-2026**
**COI**	

The authors hav attended to my (and the other reviewer's) comments in a constructive way. I have no further comments or queries. Well done!

Clarification: by asking for a table or figure displaying the steps in the analytical process, I meant a table showing chunks of quotes, and how they were coded and themes established. The authors made another interpretation of my comment (which menas it was somewhat unclear) but that does not really matter given the new version of the manuscript.

Congrats to an interesting piece of work!
